# Synthesis, Biological Evaluation, and Molecular Modeling Studies of New Oxadiazole-Stilbene Hybrids against Phytopathogenic Fungi

**DOI:** 10.1038/srep31045

**Published:** 2016-08-17

**Authors:** Weilin Jian, Daohang He, Shaoyun Song

**Affiliations:** 1 School of Chemistry and Chemical Engineering, South China University of Technology, Guangzhou, Guangdong 510640, People’s Republic of China; 2 State Key Lab of Biocontrol, Sun Yat-sen University, Guangzhou, Guangdong 510006, People’s Republic of China

## Abstract

Natural stilbenes (especially resveratrol) play important roles in plant protection by acting as both constitutive and inducible defenses. However, their exogenous applications on crops as fungicidal agents are challenged by their oxidative degradation and limited availability. In this study, a new class of resveratrol-inspired oxadiazole-stilbene hybrids was synthesized *via* Wittig-Horner reaction. Bioassay results indicated that some of the compounds exhibited potent fungicidal activity against *Botrytis cinerea in vitro*. Among these stilbene hybrids, compounds **11** showed promising inhibitory activity with the EC_50_ value of 144.6 *μ*g/mL, which was superior to that of resveratrol (315.6 *μ*g/mL). Remarkably, the considerably abnormal mycelial morphology was observed in the presence of compound **11**. The inhibitory profile was further proposed by homology modeling and molecular docking studies, which showed the possible interaction of resveratrol and oxadiazole-stilbene hybrids with the cytochrome P450-dependent sterol 14*α*-demethylase from *B. cinerea* (BcCYP51) for the first time. Taken together, these results would provide new insights into the fungicidal mechanism of stilbenes, as well as an important clue for biology-oriented synthesis of stilbene hybrids with improved bioactivity against plant pathogenic fungi in crop protection.

Stilbene-derived compounds, structurally characterized by a 1,2-diphenylethylene nucleus, constitute a unique chemical scaffold in the search for bioactive molecules. Among those stilbenes, resveratrol (Fig. 1) and its natural derivatives have attracted considerable interest both for their roles in plant defenses^1^,^2^ and for their beneficial impacts on human health^3^,^4^,^5^,^6^,^7^. Much effort dedicated to the later aspects has highlighted the health-promoting properties, one of which is associated with their chemopreventive and therapeutic effects against human cancers^3^,^4^,^5^. From a biological point of view, however, special attention should also be paid to the ecological significance of stilbenes in plant disease resistance, especially their fungitoxicity towards fungal cells.

In fact, natural stilbenes (e.g. resveratrol) appear to act as constitutive and inducible defenses in response to fungal infections such as *Botrytis cinerea*^8^,^9^,^10^,^11^, as well as to abiotic stresses^12^,^13^ and plant growth regulators^14^,^15^. Consequently, a positive correlation between stilbenes production potential and disease resistance in plants has been well established. Indeed, resveratrol and its derivatives can accumulate rapidly to high levels at site of the lesion, where the local concentrations can contribute effectively to the inhibition of fungal growth *in vitro*^1^. It is becoming increasingly clear that resveratrol has inhibitory effects on the germination of conidia and on mycelial growth of *B. cinerea*^16^,^17^,^18^. Furthermore, ultrastructural observations showed significantly cytological modifications in fungal cells, including disruption of the plasma membrane, and even a cessation of respiration in *B. cinerea* conidia, in the presence of sub-lethal or lethal concentrations of resveratrol^16^,^19^. Similar effects have also been described in *B. cinerea* treated with other resveratrol derivatives such as pterostilbene^19^,^20^. In addition to the endogenous roles of stilbenes, their exogenous applications as “natural fungicides” on fruits have been reported^21^,^22^. These findings suggest the potential of stilbenes as lead compounds for the development of effective agrochemicals; however, applications of natural stilbenes as fungicidal agents are challenged by their oxidative degradation^23^ and limited availability^24^. Further optimization of structural diversity of stilbenes to increase the potency, mainly against phytopathogenic fungi, are therefore greatly needed.

Thus far, chemical modification of natural stilbenes involves a number of strategies, including introduction of electron-withdrawing groups^23^,^25^,^26^, and hybridization with bioactive moieties^27^,^28^, replacement of the phenyl ring with heteroaromatic groups (e.g. furyl or pyridinyl groups)^29^,^30^. In particular, the approach of hybridization is becoming attractive as a modification tool in rational design of new hybrid molecules with improved bioactivity. For instance, Yan *et al*.^27^ recently reported a series of multi-target-directed benzoselenazole-stilbene hybrids that showed potent anti-proliferative activity against several cancer cell lines, indicating the cytotoxic nature of stilbene-derived hybrids. In our previous studies^31^,^32^, we introduced the 1,3,4-oxadiazole moiety into stilbene skeleton, which has led to promising results *in vivo* bioassays against *Colletotrichum lagenarium* and *Pseudoperonospora cubensis* from cucumber plants. Considering the defensive role of stilbenes in plant resistance especially against *B. cinerea*, it would be of great interest to further investigate the potential synergistic profile of oxadiazole-stilbene hybrids against this fungus compared with natural stilbenes.

In this study, we report a resveratrol-inspired synthesis of new oxadiazole-stilbene hybrids (Fig. 1), which were obtained from the Wittig-Horner reaction. Their fungicidal activities were evaluated *in vitro* against *B. cinerea*. Furthermore, the effect of the active compound on hyphal morphology of *B. cinerea* was observed. Since the fungicidal mechanism of stilbenes against fungi is not well understood, it was suggested that resveratrol could exert its fungitoxicity towards *B. cinerea*, presumably by forming protein-phenol complexes that associated with the disruption of membrane system^33^. In support of this hypothesis, we postulated the underlying interaction of resveratrol-derived stilbenes with the cytochrome P450-dependent sterol 14*α*-demethylase from *B. cinerea* (BcCYP51). In this regard, a homology model of BcCYP51 was firstly constructed using the recently reported crystal structure of *Aspergillus fumigatus* CYP51 (AfCYP51) as a template, which showed a high sequence identity (68%) with BcCYP51. Subsequently, molecular docking was carried out to predict and explain the putative binding modes of both resveratrol and stilbene hybrids with the BcCYP51. The structural information revealed from this study provides new insights into the possible molecular mechanism of the stilbenes against *B. cinerea* for the first time.

## Results and Discussion

### Synthesis

**Figure 2:**
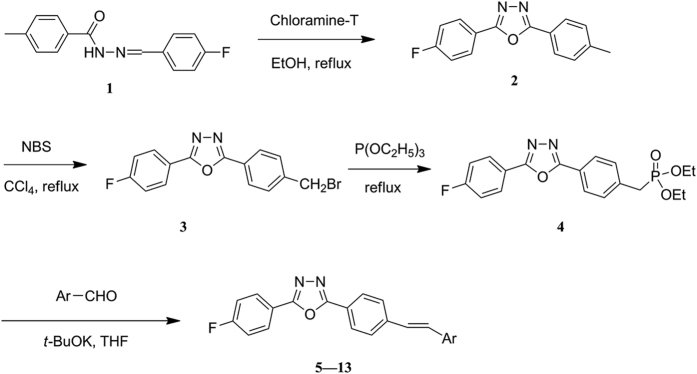
General synthetic route for the title compounds 5–13.

The synthetic route of compounds **5**–**13** is shown in Fig. 2. The new series of oxadiazole-stilbene hybrids, including two azastilbenes (**12** and **13**), was synthesized in four steps *via* oxidative cyclization of acylhydrazones, bromination of N-bromosuccinimide (NBS), and Arbuzov rearrangement followed by Wittig-Horner olefination. As indicated by ^1^H NMR, the olefinic protons (CH=CH) showed two fine doublets with a coupling constant (16.1–16.5 Hz), which were assigned to the *trans*-stilbene. All of the synthetic compounds showed appreciable spectroscopic and analytical data that were consistent with their depicted structures.

### Fungicidal Activity

*B. cinerea*, the causal agent of gray mold, is responsible for serious losses in more than 200 host species (e.g. grapes, cucurbits, and strawberries)^34^. The effect of the title compounds on the mycelial growth of plant pathogen *B. cinerea* was evaluated *in vitro*. Resveratrol was used as the positive control in the tests, and the results are summarized in Table 1. Bioassay suggested that the compounds showed moderate to promising inhibitory activities against *B. cinerea* in the initial screening test at concentration of 400 *μ*g/mL. Notably, compounds **11** and **13** exhibited potent activities with the EC_50_ values of 144.6 and 231.3 *μ*g/mL, respectively, which were superior to that of resveratrol (315.6 *μ*g/mL).

It has been reported that resveratrol at the low concentration, showed weak activity against *B. cinerea* at 48 h, whereas after 72 h of treatment it became inactive and even appeared to promote the mycelial growth^35^. Similar results have also been observed for the bioactivities of resveratrol in our study. It was suggested that the inducible detoxification mechanism may play important role in the pathogen-phytoalexin (stilbene) interactions^35^. Indeed, the metabolism of stilbene phytoalexin could be related to the pathogenicity of *B. cinerea*^36^. In contrast to the oxadiazole-stilbene hybrids, however, no such phenomenon was observed during assay time. On the basis of the results, it may be concluded that structural modification of natural stilbene by hybridization with oxadiazole, particularly replacement of one phenyl ring with heteroaromatic groups (**11**, **12**, and **13**), was showed to be an efficient strategy in finding new lead structures for plant disease control.

### Effect on Hyphal Morphology of *B. cinerea*

**Figure 3:**
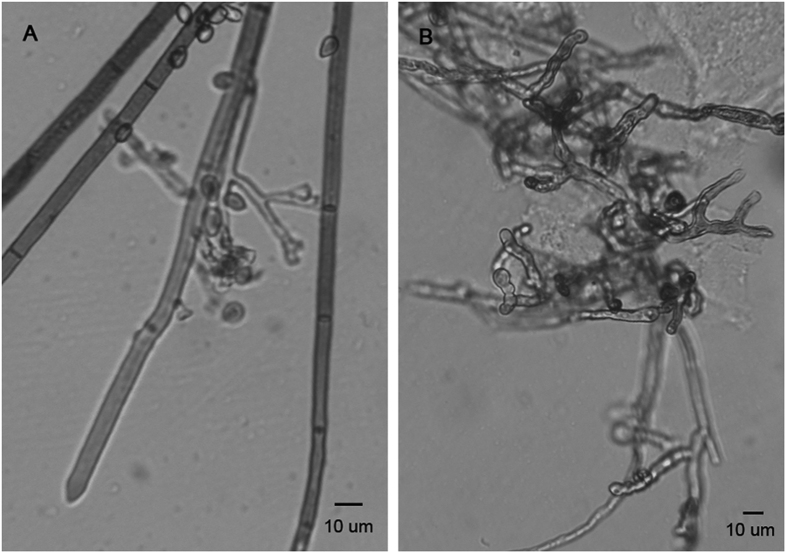
Microscopic observation of hyphal morphology of (**A**) *B. cinerea* from the control and (**B**) cultures treated with compound **11** showing deformed mycelia of *B. cinerea*.

The effect on the mycelia of *B. cinerea* was observed with a microscope. Microscopic observation showed considerably modified mycelial morphology in the presence of **11** (Fig. 3B). The hyphae were distorted with constricted structures compared with the control (Fig. 3A). The results were consistent with our previous study in which the mycelial cell membrane system was significantly damaged by the membrane permeability assay^32^.

### Interactions Between CYP51 and Stilbenes

Despite the membrane-disruption effects of resveratrol and oxadiazole-stilbene hybrids on fungal cells, their mode of action was not well elucidated at a molecular level. Early studies suggested that resveratrol could exert its fungitoxicity presumably by forming protein-phenol complexes, which were associated with the disruption of membrane system^33^. Such effects were further supposed be linked to the inhibition of ergosterol biosynthesis^37^. Cytochromes P450 (CYPs) play crucial roles in primary and secondary metabolic pathways, as well as in the metabolism of numerous xenobiotics including pesticides^38^. Among the fungal P450s family, sterol 14*α*-demethylase (CYP51), generally catalyzing a key step in the biosynthesis of membrane ergosterol, is the primary target of antifungal agents^39^. Moreover, the catalytic potential of fungal CYP51^40^ and human CYPs family^41^,^42^ in bioconversion of stilbene derivatives has been well documented. It is therefore reasonable to postulate the possible interactions between the stilbenes and CYP51 enzyme.

#### Homology modeling

**Figure 4:**
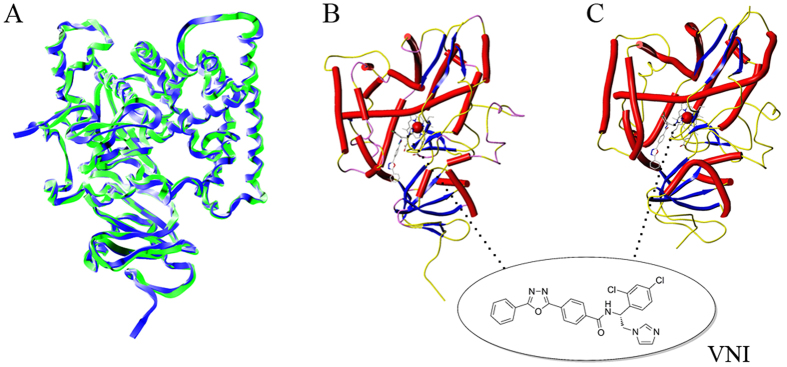
Ribbon diagram of the BcCYP51 homology model and the template: (**A**) superimposition of the model (blue) and template (green), and secondary structures of the mode (**B**) and the template (**C**) in complex with VNI.

To verify our hypothesis, we carried out molecular modeling of CYP51 from *B. cinerea* (BcCYP51). Nevertheless, the structural information on three-dimensional (3D) mode of BcCYP51 remained sparse. A previous docking study constructed the homology mode of BcCYP51 on the basis of the crystal structure CYP from *Mycobacterium tuberculosis* (MtCYP51), which showed only low sequence identity (<30%) with BcCYP51 enzyme^43^. Until recently, the crystal structure of *Aspergillus fumigatus* CYP51 (AfCYP51) complexed with inhibitor VNI ((*R*)-*N*-(1-(2,4-dichlorophenyl)-2-(1*H*-imidazol-1-yl)ethyl)-4-(5- phenyl-1,3,4-oxadiazol-2-yl)benzamide) was reported in 2015^44^. Due to the structural similarity between the co-crystallized VNI and the studied compounds, and the high sequence identity (68%), we firstly constructed the mode of BcCYP51 using the crystal structure of AfCYP51 as a template. The minimized mode was superimposed with the template to compare the secondary structure of the protein CYP51 (Fig. 4). Evaluation of the homology mode by Ramachandran plot (see Supplementary Fig. S1) showed that >99% residues were located in the allowed regions. The only two disallowed residues were Val61 and Val135, which were irrelevant to the active sites. The results indicated the reliable stereochemical quality of the homology mode.

#### Docking Mode Analysis

**Figure 5:**
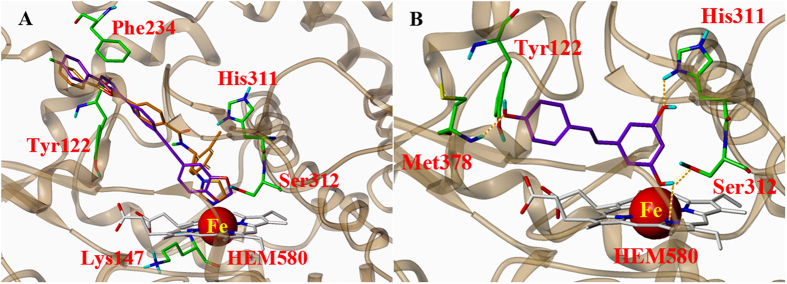
Docking representation of the binding modes to the BcCYP51 of (**A**) compound **11** (purple) overlapped with VNI (yellow), and (**B**) resveratrol in sticks colored by atom type. Heme is shown as gray sticks, and key residues are represented with green sticks.

Molecular docking of compounds **6**, **11**, **13**, and resveratrol into the active site of BcCYP51 was performed with Surflex-Dock module in the Sybyl. To elucidate the possible protein-ligand interactions, the detailed docking modes of the active compound **11** and resveratrol are shown in Fig. 5. The putative docking pose of **11** was overlapped with that of co-crystallized VNI (Fig. 5A). Consistent with the binding mode of VNI, no H-bond was formed with the protein. However, the hydrophobic and van der Waals interactions between **11** and surrounding residues (e.g. Leu92, Tyr122, Lys147, Met235) were observed in the hydrophobic pocket. In particular, the oxadiazole ring forms a *π*–*π* stacking interaction with Phe234, which were suggested to be crucial in stabilizing the preferred orientation of ligands in the active site pocket^44^. Interestingly, one oxygen atom of the benzodiozole ring was direct towards heme iron with a distance of 2.1 Å.

In comparison with **11**, resveratrol had a different binding mode with the protein (Fig. 5B). H-bonding analysis showed that four hydrogen bonds were formed between resveratrol and the residues His311, Ser312, Met378, and Heme. Consequently, the 3-hydroxy group involved in hydrogen binding with Ser312 and Heme made the molecule come closer to the heme iron (the distance was 1.9 Å). However, no *π*–*π* stacking interactions were formed in the binding mode. The docking result also showed the reduced hydrophobic interactions, which may account for its fair inhibitory activity. It was suggested that the potency of resveratrol could be related to its less hydrophobicity that limits diffusion across the cytoplasmic membrane^1^. In line with these findings, indirect evidence revealed the positive correlation between the binding affinity of hydroxyl stilbenes with human CYPs and their lipophilicity^45^. The docking results indicate that the patterns of stilbene skeleton (different substituents, replacement of heterocyclic rings) are essential determinants of ligand affinity, which may account for their *in vitro* inhibitory activity.

Recently, combretastatin A-4 (a *cis*-stilbene) was postulated as a potential fungicide targeting fungal tubulin^46^. Contrary to the combretastatin A-4 derivatives, *trans*-stilbenes were showed to bind with tubulin, but could not inhibit microtubule assembly^47^. In other words, *trans*-stilbenes are likely to interact with a different target site. Nevertheless, our findings, together with the previous studies, showed the possible interactions of *trans*-stilbenes with fungal CYP51 protein. The information revealed from this study would also provide a new starting point for chemical modification of natural *trans*-stilbenes, and could shed lights on the precise information on protein-ligand interactions. One might expect such information from the enzyme inhibition assay combined with binding mode and crystallographic analysis. Such endeavors are in progress in our research group.

## Materials and Methods

### Chemicals and Instruments

All chemicals and reagents were commercially available and used without further purification. All solvents were dried and redistilled prior to use. Melting points were determined on an SGW X-4 microscope melting point apparatus (Shanghai Instrument Physical Optics Instrument Co. Ltd., Shanghai, China) and were uncorrected. ^1^H and ^13^C nuclear magnetic resonance (NMR) spectra were recorded in CDCl_3_ or DMSO-*d*_6_ on a Bruker AV-600 MHz NMR spectrometer using tetramethylsilane (TMS) as an internal standard. High resolution mass spectra (HRMS) were obtained with a Bruker maXis impact spectrometer [electrospray ionization (ESI)]. The purity of the compounds was confirmed by thin-layer chromatography (TLC) on silica gel “G”-coated glass plates, and spots were visualized under ultraviolet (UV) irradiation.

### Pathogens and Cultures

*Botrytis cinerea* Pers. was provided by Hunan Research Institute of Chemical Industry, National Engineering Research Center for Agrochemicals (Changsha, China). After retrieval from the storage tube, the strains were incubated on potato dextrose agar (PDA) and maintained at 21 °C with a 12-h light photoperiod.

### Synthetic Procedures

Intermediates **1**–**4** were synthesized according to our previously reported procedures^32^. The data for compounds **5**–**13** are shown below.

#### (E)-2-(4-Fluorophenyl)-5-(4-(2-methoxystyryl)phenyl)-1,3,4-oxadiazole **5**

Light green solid; yield, 65.1%; mp, 134–135 °C; ^1^H NMR (600 MHz, CDCl_3_), δ 8.16 (dd, *J* = 8.7, 5.3 Hz, 2H, C_6_H_4_ 2,6-H), 8.10 (d, *J* = 8.2 Hz, 2H, C_6_H_4_ 2,6-H), 7.68 (d, *J* = 8.2 Hz, 2H, C_6_H_4_ 3,5-H), 7.62 (dd, *J* = 16.2, 7.2 Hz, 2H, CH=CH, C_6_H_4_ 6H), 7.33 – 7.28 (m, 1H, C_6_H_4_ 4H), 7.24 (t, *J* = 8.5 Hz, 2H, C_6_H_4_ 3,5-H), 7.16 (d, *J* = 16.5 Hz, 1H, CH=CH), 7.00 (t, *J* = 7.5 Hz, 1H, C_6_H_4_ 5-H), 6.94 (d, *J* = 8.2 Hz, 1H, C_6_H_4_ 3-H), 3.93 (s, 3H, OCH_3_); ^13^C NMR (151 MHz, CDCl_3_), δ 165.60, 164.60, 163.92, 163.60, 157.18, 141.53, 129.34, 129.20, 129.14, 127.74, 127.17, 127.03, 126.70, 126.03, 125.81, 122.17, 120.80, 120.37, 120.35, 116.45, 116.30, 111.04, 55.53; HRMS (ESI), *m/z* calcd for C_23_H_18_FN_2_O_2_ [M + H]^+^ 373.1347; found, 373.1347.

#### (E)-2-(4-Fluorophenyl)-5-(4-(3-methoxystyryl)phenyl)-1,3,4-oxadiazole **6**

Light green solid; yield, 76.5%; mp, 182–183 °C; ^1^H NMR (600 MHz, CDCl_3_), δ 8.20 – 8.15 (m, 2H, C_6_H_4_ 2,6-H), 8.12 (d, *J* = 8.0 Hz, 2H, C_6_H_4_ 2,6-H), 7.67 (d, *J* = 8.0 Hz, 2H, C_6_H_4_ 3,5-H), 7.32 (t, *J* = 7.9 Hz, 1H, C_6_H_4_ 5-H), 7.25 (t, *J* = 8.4 Hz, 2H, C_6_H_4_ 3,5-H), 7.22 (d, *J* = 16.1 Hz, 1H, CH=CH), 7.17 (d, *J* = 6.9 Hz, 1H, C_6_H_4_ 6-H), 7.14 (d, *J* = 16.2 Hz, 1H, CH=CH), 7.09 (s, 1H, C_6_H_4_ 2-H), 6.91 – 6.85 (m, 1H, C_6_H_4_ 4-H), 3.88 (s, 3H, OCH_3_); ^13^C NMR (101 MHz, CDCl_3_), δ 166.05, 164.49, 163.67, 163.54, 159.98, 140.70, 138.15, 130.98, 129.76, 129.24, 129.15, 127.65, 127.24, 127.05, 122.53, 120.34, 120.30, 119.51, 116.52, 116.30, 113.95, 112.08, 55.28; HRMS (ESI), *m/z* calcd for C_23_H_18_FN_2_O_2_ [M + H]^+^ 373.1347; found, 373.1347.

#### (E)-2-(4-(2-Chlorostyryl)phenyl)-5-(4-fluorophenyl)-1,3,4-oxadiazole **7**

Light green solid; yield, 67.4%; mp, 165–167 °C; ^1^H NMR (600 MHz, CDCl_3_), δ 8.18 – 8.13 (m, 2H, C_6_H_4_ 2,6-H), 8.13 – 8.10 (m, 2H, C_6_H_4_ 2,6-H), 7.70 (dd, *J* = 7.9, 1.6 Hz, 1H, C_6_H_4_ 3-H), 7.69 (d, *J* = 8.2 Hz, 2H, C_6_H_4_ 3,5-H), 7.63 (d, *J* = 16.3 Hz, 1H, CH=CH), 7.42 (dd, *J* = 7.9, 1.3 Hz, 1H, C_6_H_4_ 6-H), 7.30 (td, *J* = 7.4, 0.9 Hz, 1H, C_6_H_4_ 5-H), 7.27 – 7.21 (m, 3H, C_6_H_4_ 3,5-H, C_6_H_4_ 4-H), 7.11 (d, *J* = 16.3 Hz, 1H, CH=CH); ^13^C NMR (151 MHz, CDCl_3_), δ 165.65, 164.42, 163.97, 163.72, 140.47, 134.83, 133.76, 129.95, 129.88, 129.23, 129.18, 129.12, 127.35, 127.27, 127.05, 127.01, 126.60, 122.92, 120.31, 120.28, 116.49, 116.34; HRMS (ESI), *m/z* calcd for C_22_H_15_ClFN_2_O [M + H]^+^ 377.0851; found, 377.0851.

#### (E)-2-(4-(2-Bromostyryl)phenyl)-5-(4-fluorophenyl)-1,3,4-oxadiazole **8**

Light green solid; yield, 66.3%; mp, 198–200 °C; ^1^H NMR (600 MHz, CDCl_3_), δ 8.17 – 8.14 (m, 2H, C_6_H_4_ 2,6-H), 8.12 (d, *J* = 8.3 Hz, 2H, C_6_H_4_ 2,6-H), 7.69 (d, *J* = 8.2 Hz, 3H, C_6_H_4_ 3,5-H, C_6_H_4_ 3-H), 7.62 (d, *J* = 1.0 Hz, 1H, C_6_H_4_ 6-H), 7.59 (d, *J* = 16.5 Hz, 1H, CH=CH), 7.34 (t, *J* = 7.3 Hz, 1H, C_6_H_4_ 5-H), 7.26 – 7.21 (m, 2H, C_6_H_4_ 3,5-H), 7.16 (td, *J* = 7.9, 1.5 Hz, 1H, C_6_H_4_ 4-H), 7.06 (d, *J* = 16.2 Hz, 1H, CH=CH); ^13^C NMR (151 MHz, CDCl_3_), δ 165.63, 164.40, 163.95, 163.71, 140.38, 136.51, 133.20, 130.04, 129.70, 129.37, 129.23, 129.17, 127.64, 127.35, 127.27, 126.81, 124.41, 122.91, 120.28, 120.26, 116.49, 116.35; HRMS (ESI), *m/z* calcd for C_22_H_14_BrFN_2_NaO [M + Na]^+^ 443.0166; found, 443.0165.

#### (E)-2-(4-Fluorophenyl)-5-(4-(2-nitrostyryl)phenyl)-1,3,4-oxadiazole **9**

Yellow solid; yield, 68.5%; mp, 205–206 °C; ^1^H NMR (600 MHz, CDCl_3_), δ 8.16 (dd, *J* = 8.6, 5.3 Hz, 2H, C_6_H_4_ 2,6-H), 8.14 (d, *J* = 8.2 Hz, 2H, C_6_H_4_ 2,6-H), 8.01 (d, *J* = 8.1 Hz, 1H, C_6_H_4_ 3-H), 7.79 (d, *J* = 7.8 Hz, 1H, C_6_H_4_ 6-H), 7.73 (d, *J* = 16.1 Hz, 1H, CH=CH), 7.69 (d, *J* = 8.2 Hz, 2H, C_6_H_4_ 3,5-H), 7.65 (t, *J* = 7.5 Hz, 1H, C_6_H_4_ 5-H), 7.46 (t, *J* = 7.7 Hz, 1H, C_6_H_4_ 4-H), 7.24 (t, *J* = 8.5 Hz, 2H, C_6_H_4_ 3,5-H), 7.11 (d, *J* = 16.1 Hz, 1H, CH=CH); ^13^C NMR (151 MHz, CDCl_3_), δ 165.68, 164.32, 164.00, 163.81, 148.10, 139.84, 133.23, 132.50, 132.40, 129.27, 129.21, 128.54, 128.28, 127.63, 127.33, 125.95, 124.90, 123.45, 120.25, 120.23, 116.52, 116.37; HRMS (ESI), *m/z* calcd for C_22_H_15_FN_3_O_3_ [M + H]^+^ 388.1092; found, 388.1092.

#### Sodium (E)-4-(4-(5-(4-fluorophenyl)-1,3,4-oxadiazol-2-yl)styryl)benzene-1,3- disulfonate **10**

Dark yellow solid; yield, 42.2%; mp >300 °C; ^1^H NMR (600 MHz, DMSO-*d*_6_), δ 8.21 – 8.17 (m, 3H, C_6_H_4_ 2,6-H, C_6_H_3_ 3-H), 8.10 (d, *J* = 1.5 Hz, 1H, C_6_H_3_ 5-H), 7.94 (d, *J* = 8.1 Hz, 2H, C_6_H_4_ 2,6-H), 7.73 – 7.67 (m, 2H, C_6_H_3_ 6-H, CH=CH), 7.48 (d, *J* = 8.6 Hz, 2H, C_6_H_4_ 3,5-H), 7.47 – 7.46 (m, 3H, C_6_H_4_ 3,5-H, CH=CH); ^13^C NMR (151 MHz, DMSO), δ 169.70, 168.49, 168.16, 157.37, 154.95, 137.51, 135.57, 134.56, 134.51, 131.54, 131.12, 131.10, 129.70, 125.42, 124.73, 121.93, 121.78; HRMS (ESI), *m/z* calcd for C_22_H_13_FN_2_Na_3_O_7_S_2_ [M + Na]^+^ 568.9836; found, 568.9837.

#### (E)-2-(4-(2-(Benzo[d][1,3]dioxol-5-yl)vinyl)phenyl)-5-(4-fluorophenyl)-1,3,4-oxadiazole **11**

Light green solid; yield, 74.1%; mp, 213–214 °C; ^1^H NMR (600 MHz, CDCl_3_), δ 8.18 – 8.13 (m, 2H, C_6_H_4_ 2,6-H), 8.09 (d, *J* = 8.2 Hz, 2H, C_6_H_4_ 2,6-H), 7.61 (d, *J* = 8.2 Hz, 2H, C_6_H_4_ 3,5-H), 7.24 (t, *J* = 8.5 Hz, 2H, C_6_H_4_ 3,5-H), 7.14 (d, *J* = 16.2 Hz, 1H, CH=CH), 7.09 (s, 1H, C_6_H_3_ 2-H), 6.98 (d, *J* = 8.3 Hz, 1H, C_6_H_3_ 6-H), 6.96 (d, *J* = 16.5 Hz, 1H, CH=CH), 6.83 (d, *J* = 8.0 Hz, 1H, C_6_H_3_ 5-H), 6.00 (s, 2H, CH_2_); ^13^C NMR (151 MHz, CDCl_3_), δ 165.63, 164.55, 163.95, 163.63, 148.30, 147.92, 140.95, 131.27, 130.77, 129.21, 129.15, 127.24, 126.78, 125.64, 122.21, 122.09, 120.37, 120.34, 116.47, 116.32, 108.51, 105.66, 101.27; HRMS (ESI), *m/z* calcd for C_23_H_16_FN_2_O_3_ [M + H]^+^ 387.1139; found, 387.1139.

#### (E)-2-(4-Fluorophenyl)-5-(4-(2-(pyridin-3-yl)vinyl)phenyl)-1,3,4-oxadiazole **12**

Light green solid; yield, 86.4%; mp, 174–176 °C; ^1^H NMR (600 MHz, CDCl_3_), δ 8.76 (s, 1H, pyridine-H), 8.53 (dd, *J* = 2.8, 1.7 Hz, 1H, pyridine-H), 8.17 – 8.14 (m, 2H, C_6_H_4_ 2,6-H), 8.12 (dd, *J* = 8.4, 1.8 Hz, 2H, C_6_H_4_ 3,5-H), 7.86 (d, *J* = 6.6 Hz, 1H, pyridine-H), 7.69 – 7.65 (m, 2H, C_6_H_4_ 2,6-H), 7.32 (dd, *J* = 6.5, 6.0 Hz, 1H, pyridine-H), 7.23 (td, *J* = 8.6, 1.6 Hz, 2H, C_6_H_4_ 3,5-H), 7.19 (s, 2H, CH=CH); ^13^C NMR (151 MHz, CDCl_3_), δ 165.65, 164.33, 163.97, 163.74, 149.04, 148.65, 140.00, 132.94, 132.44, 129.49, 129.22, 129.16, 127.30, 127.23, 127.19, 123.61, 123.10, 120.26, 120.24, 116.49, 116.34; HRMS (ESI), *m/z* calcd for C_21_H_15_FN_3_O [M + H]^+^ 344.1194; found, 344.1194.

#### (E)-2-(4-Fluorophenyl)-5-(4-(2-(pyridin-4-yl)vinyl)phenyl)-1,3,4-oxadiazole **13**

Light green solid; yield, 75.1%; mp, 202–203 °C; ^1^H NMR (600 MHz, CDCl_3_), δ 8.60 (d, *J* = 4.9 Hz, 2H, pyridine-H), 8.17 – 8.12 (m, 2H, C_6_H_4_ 2,6-H), 8.11 (d, *J* = 8.0 Hz, 2H, C_6_H_4_ 3,5-H), 7.66 (d, *J* = 8.1 Hz, 2H, C_6_H_4_ 2,6-H), 7.38 (d, *J* = 4.9 Hz, 2H, pyridine-H), 7.30 (d, *J* = 16.3 Hz, 1H, CH=CH), 7.22 (t, *J* = 8.4 Hz, 2H, C_6_H_4_ 3,5-H), 7.11 (d, *J* = 16.3 Hz, 1H, CH=CH); ^13^C NMR (151 MHz, CDCl_3_), δ 165.67, 164.23, 163.99, 163.80, 150.31, 143.93, 139.47, 131.74, 129.24, 129.18, 128.68, 128.29, 127.55, 127.31, 127.18, 123.55, 120.95, 120.20, 120.18, 116.51, 116.36; HRMS (ESI), *m/z* calcd for C_21_H_15_FN_3_O [M + H]^+^ 344.1194; found, 344.1195.

### *In Vitro* Bioassays

The *in vitro* fungicidal activity against *B. cinerea* was tested using the mycelial growth inhibition method^48^. The tested compounds were dissolved in dimethyl sulfoxide (DMSO) and diluted with distilled water containing 0.05% Tween 80 to prepare the 10 mg/mL stock solution. The resulting solution was mixed aseptically with molten PDA at 45–50 °C and was then distributed equally into 90 mm Petri dishes (15 mL∙dish^−1^) to produce the toxic culture medium (containing 0.5% DMSO). Mycelial discs (5mm in diameter) removed from the margins of actively growing colonies of mycelium were placed in the center area of each plate. The 0.5% (v/v) of DMSO in sterile distilled water was used as a blank control, while the resveratrol (HPLC purity ≥98%, Shanghai Yuanye Bio-Techenology Co., Ltd., Shanghai) was set as the positive control. Each treatment consisted of at least three replicates.

After 72 hours of incubation at 25 ± 2 °C, the mycelial growth diameters (in mm) were measured. The inhibition percentages were calculated *via* the following equation (1):


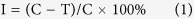


where I the rate of inhibition (%), T is the mycelial diameter (mm) in Petri dishes with compounds, and C is the diameter (mm) of the blank control. Results were expressed as the half maximal effective concentration (EC_50_), determined by regressing the inhibition of radial growth values (percent control) against the values of compound concentration. The EC_50_ values were computed from at least three separate analyses of growth inhibition using the software package SPSS v. 20.0.

### Effect on Hyphal Morphology of *B. cinerea*

To elucidate the effect on hyphal morphology alterations with the active stilbene hybrids, the mycelia of *B. cinerea* taken from areas showing the strong inhibitory level were placed on the slides and observed under a light microscope. A sample processed similarly with 0.5% of DMSO was set as the control^32^.

### Homology Modeling

The amino acid sequence of *B. cinerea* CYP51 (accession number: AAF85983) was taken from the NCBI protein database (http://www.ncbi.nlm.nih.gov/protein). A crystal structure of *Aspergillus fumigatus* CYP51 (PDB code 4UYL) was used as the crystallographic coordinate template. Homology modeling of CYP51 from *B. cinerea* was performed based on the reference protein model using FUGUE and ORCHESTRAR module integrated in Sybyl-X 2.0^49^. The optimized model was evaluated by the Ramachandran plot analysis for molecular docking.

### Molecular Docking

The automatic docking was carried out using the Surflex-Dock module implemented in the Sybyl program. During the docking procedures, water molecules and ligands were removed from the protein. Resveratrol and oxadiazole-stilbene hybrids were constructed using the 2D sketcher module in Sybyl. All ligand structures were minimized to obtain the minimum energy conformations with the Minimize module of Sybyl. Minimization was achieved using the steepest descent method for the first 100 steps, and was terminated when the root mean square deviation (RMSD) of the gradient reached a maximum cut-off of 0.005 kcal/(mol·Å). Other algorithms and parameters were set as default. The studied ligands were then docked into the active site of the BcCYP51, and their binding poses were analyzed by a scoring function and a patented search engine in Surflex-Dock.

## Additional Information

**How to cite this article**: Jian, W. *et al*. Synthesis, Biological Evaluation, and Molecular Modeling Studies of New Oxadiazole-Stilbene Hybrids against Phytopathogenic Fungi. *Sci. Rep.* **6**, 31045; doi: 10.1038/srep31045 (2016).

## Supplementary Material

Supplementary Information

## Figures and Tables

**Figure 1:**
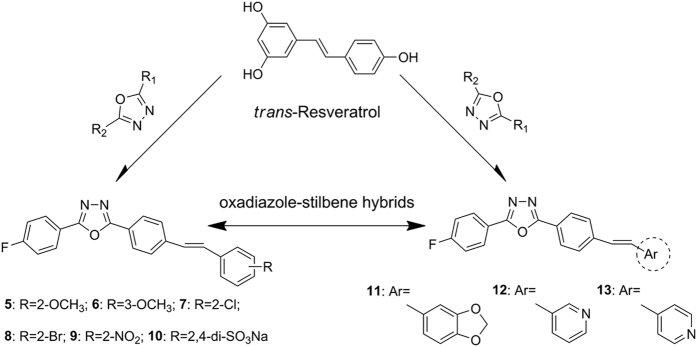
Design strategy for oxadiazole-stilbene hybrids and chemical structures of compounds 5–13 and resveratrol.

**Table 1:**
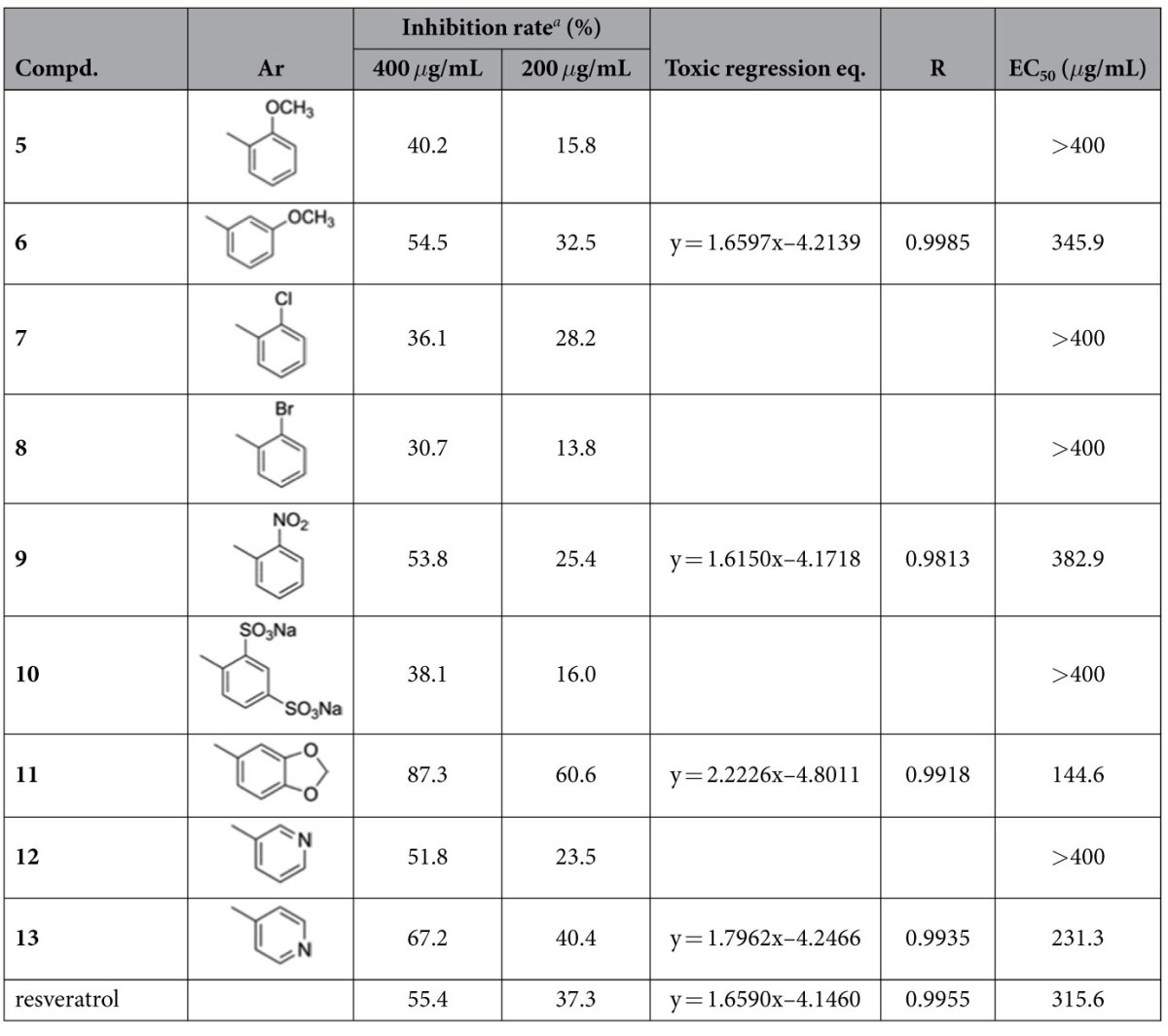
*In Vitro* Fungicidal Activity and Toxicity of the Tested Compounds Against *B. cinerea*.

^*a*^Inhibition rate of mycelial growth is based on the average colony diameter measured after 72 h of incubation. Each point represents the mean of at least three independent experiments.
