# Supplementary material for: Synthesis, Biological Evaluation, and Molecular Modeling Studies of New Oxadiazole-Stilbene Hybrids against Phytopathogenic Fungi
Source: Sci Rep. 2016 Aug 17;6:31045. doi: 10.1038/srep31045 (PMC4987640; doi:10.1038/srep31045)
Supplement: Supplementary Information [file srep31045-s1.pdf]

Synthesis, Biological Evaluation, and Molecular Modeling Studies of New Oxadiazole-Stilbene Hybrids against Phytopathogenic Fungi

Weilin Jian,[1] Daohang He,[1,*] & Shaoyun Song[2]

[1] School of Chemistry and Chemical Engineering, South China University of Technology, Guangzhou, Guangdong 510640, People's Republic of China

[2] State Key Lab of Biocontrol, Sun Yat-sen University, Guangzhou, Guangdong 510006, People's Republic of China

[*]Corresponding author, Telephone/Fax: + 86-20- 8711 -0234;

E-mail: he16221@163.com.

Contents

1. General synthetic procedures for title compounds **5**–**13**. S2

2. Mode validation by Ramachandran plot (Figure S1) S3

3. ^{1}H-NMR and ^{13}C-NMR spectra of compounds **5**–**13**. S4-S11

4. References. S12

Synthetic procedures. The desired starting materials were prepared by esterification of 4-methylbenzoic acid, followed by treatment with hydrazine hydrate in absolute ethanol. Intermediate **1** was prepared by the condensation of 4-methylbenzohydrazide and 4-fluorobenzaldehyde in an ethanol solution. Intermediates **2**–**4** were synthesized according to our previously reported procedure.[1,2]

General Procedure for Preparation of Title Compounds 5–13. To a stirred solution of the aromatic aldehyde (2 mmol) and intermediate **4** (2 mmol) in anhydrous THF (15 mL) under nitrogen atmosphere was added dropwise a solution of t-BuOK (3 mmol) in 5 mL of ethanol. The resulting mixture was stirred overnight at room temperature and then filtered and washed with ethanol. The residue was recrystallized from ethanol/DMSO to afford the corresponding oxadiazole-stilbene hybrids **5**–**13**.

Mode validation by Ramachandran plot. The stereochemical quality of the homology mode was evaluated by Ramachandran plot using PROCHECK approach.[3] In the BcCYP51 mode, 99.5% residues were located in the allowed regions, and only 0.5% residues (Val61 and Val135) were located in the disallowed regions.

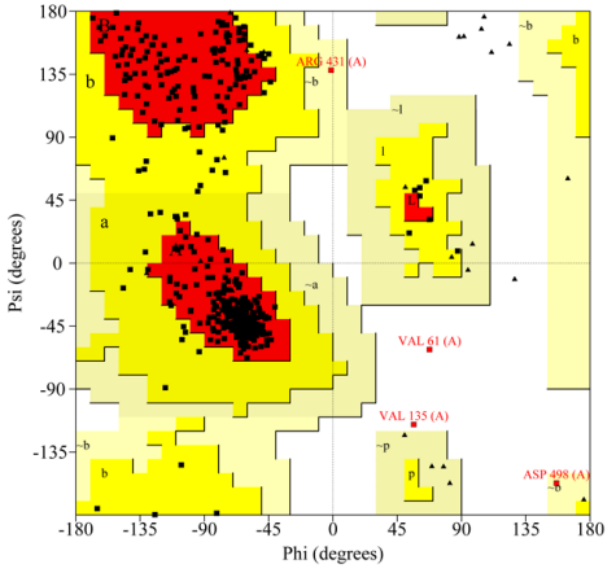

Figure S1. The Ramachandran plot of BcCYP51.

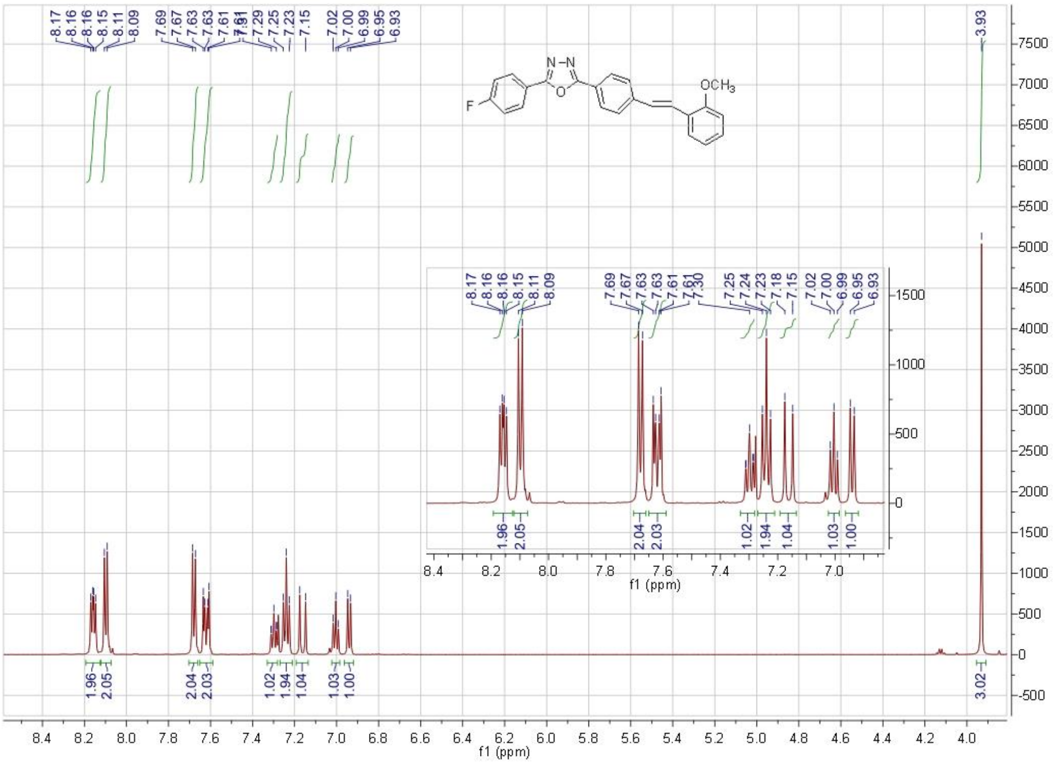

Figure S2-1. ^{1}H NMR spectrum of compound **5**.

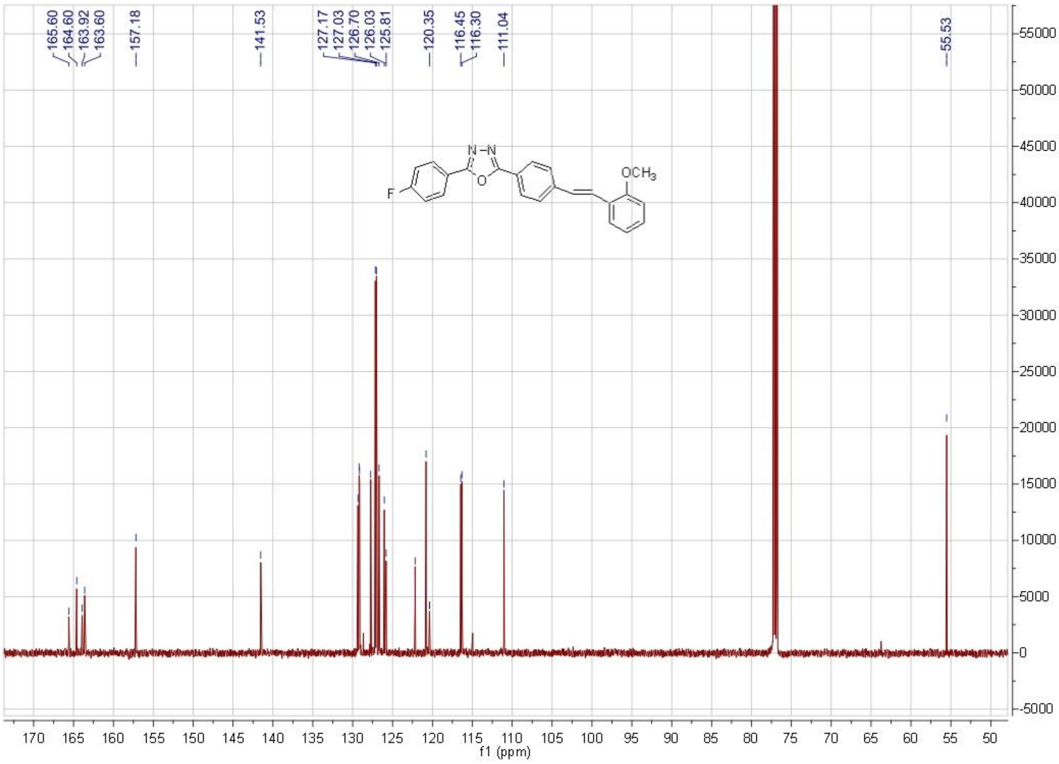

Figure S2-2. ^{13}C NMR spectrum of compound **5**.

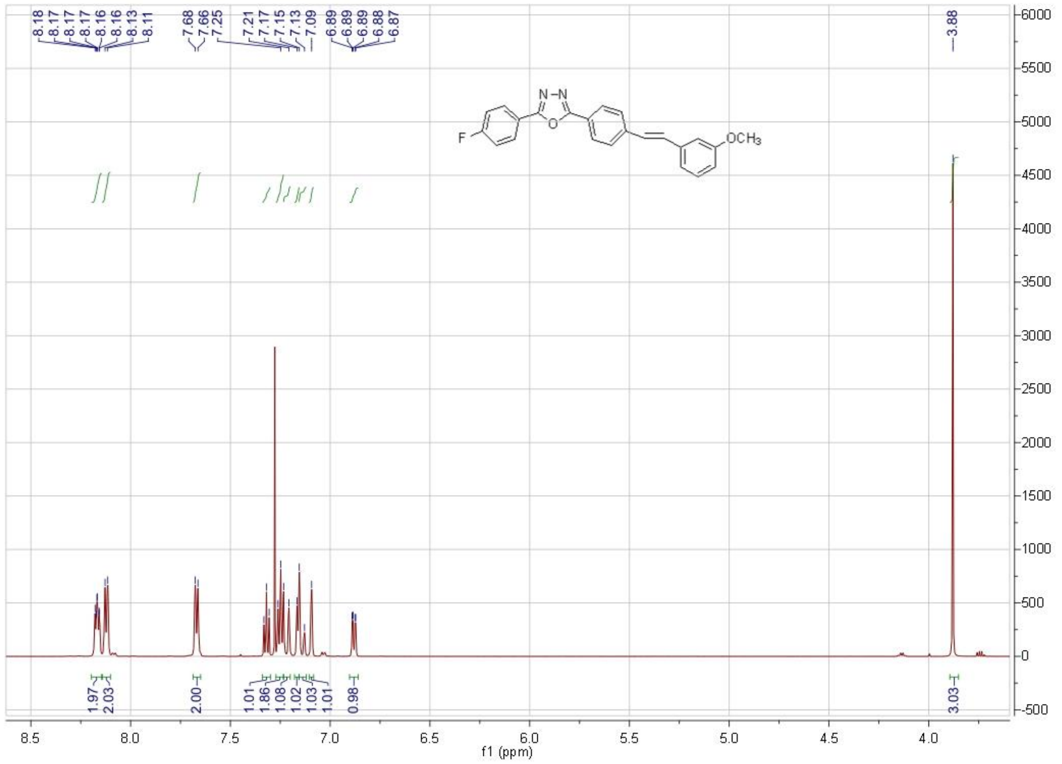

Figure S3-1. ^{1}H NMR spectrum of compound **6**.

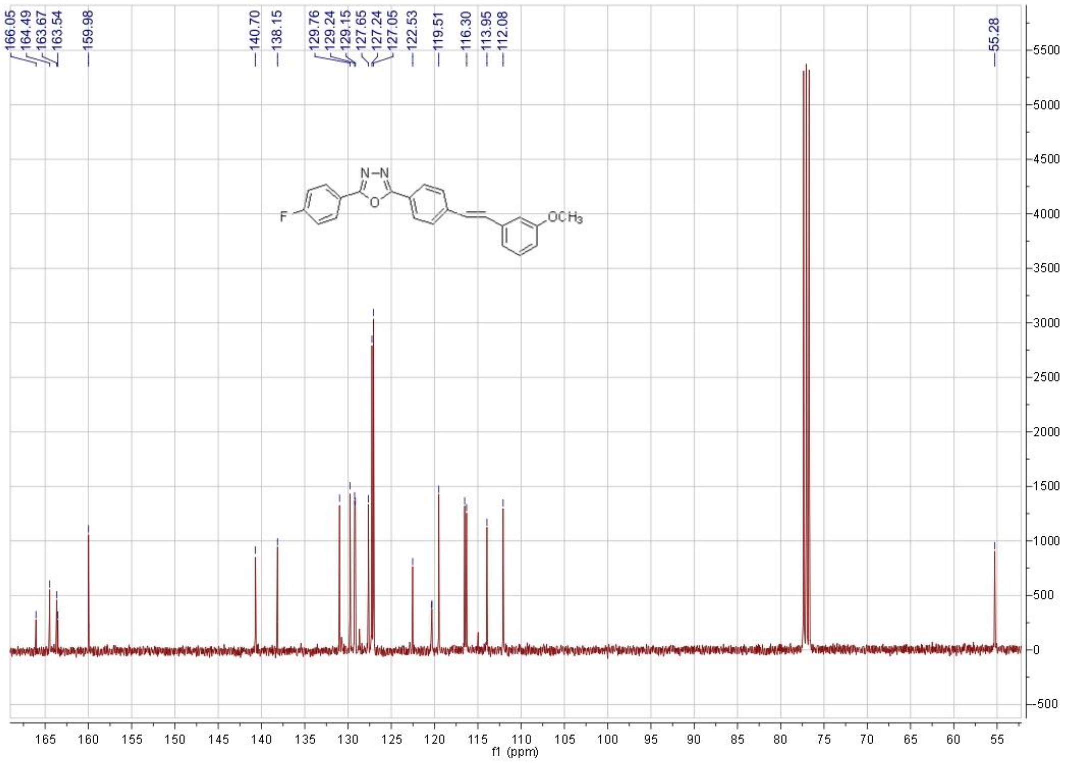

Figure S3-2. ^{13}C NMR spectrum of compound **6**.

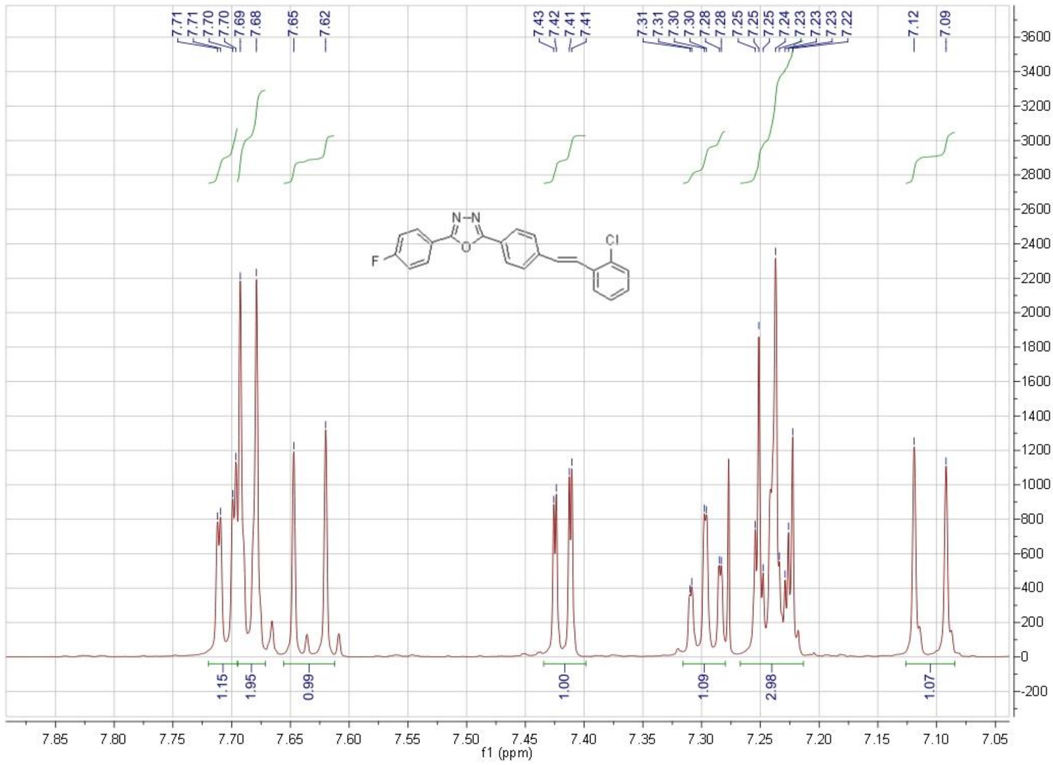

Figure S4-1. ^{1}H NMR spectrum of compound **7**.

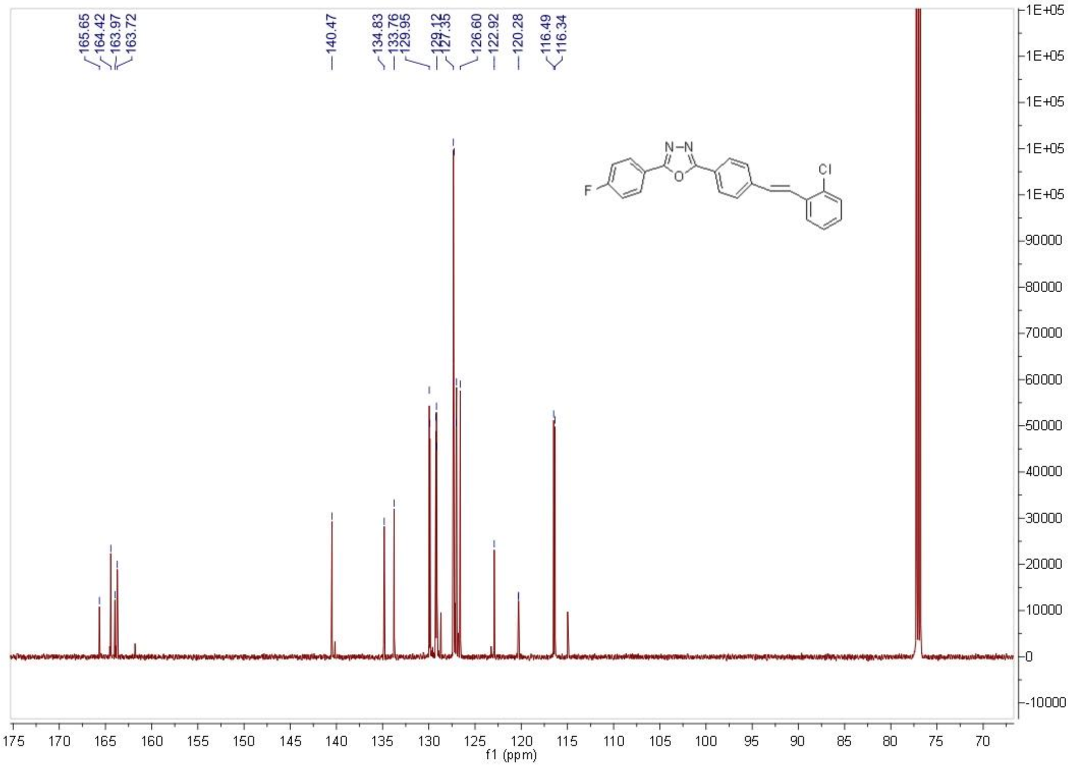

Figure S4-2. ^{13}C NMR spectrum of compound **7**.

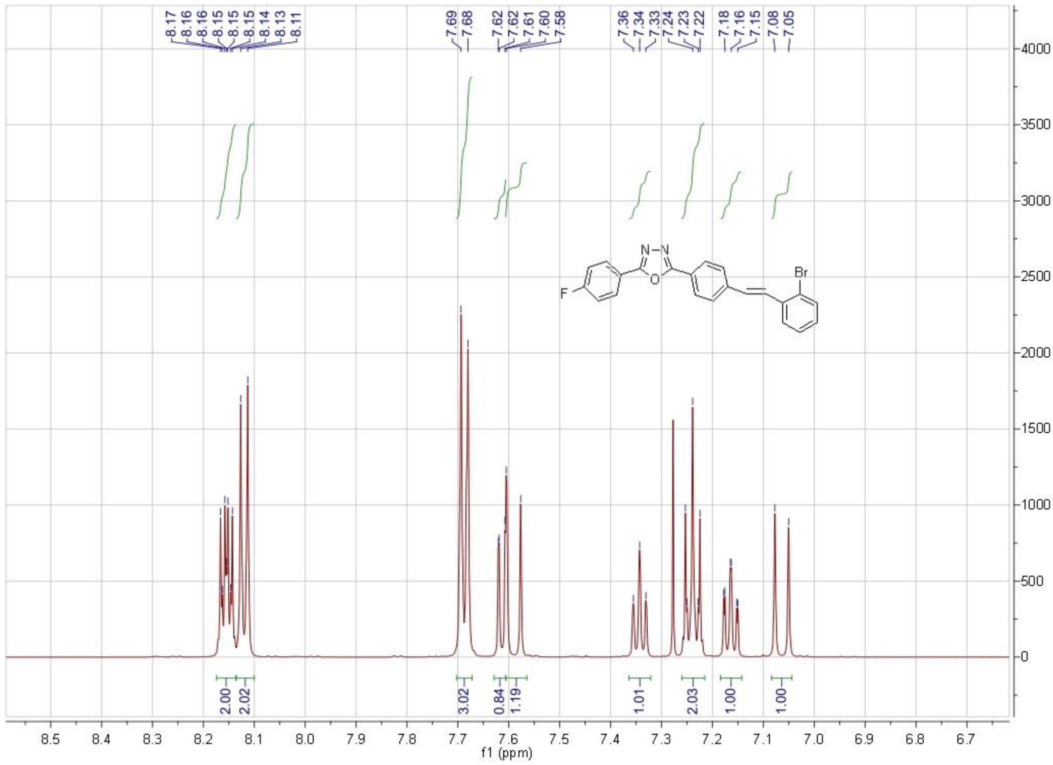

Figure S5-1. ^{1}H NMR spectrum of compound **8**.

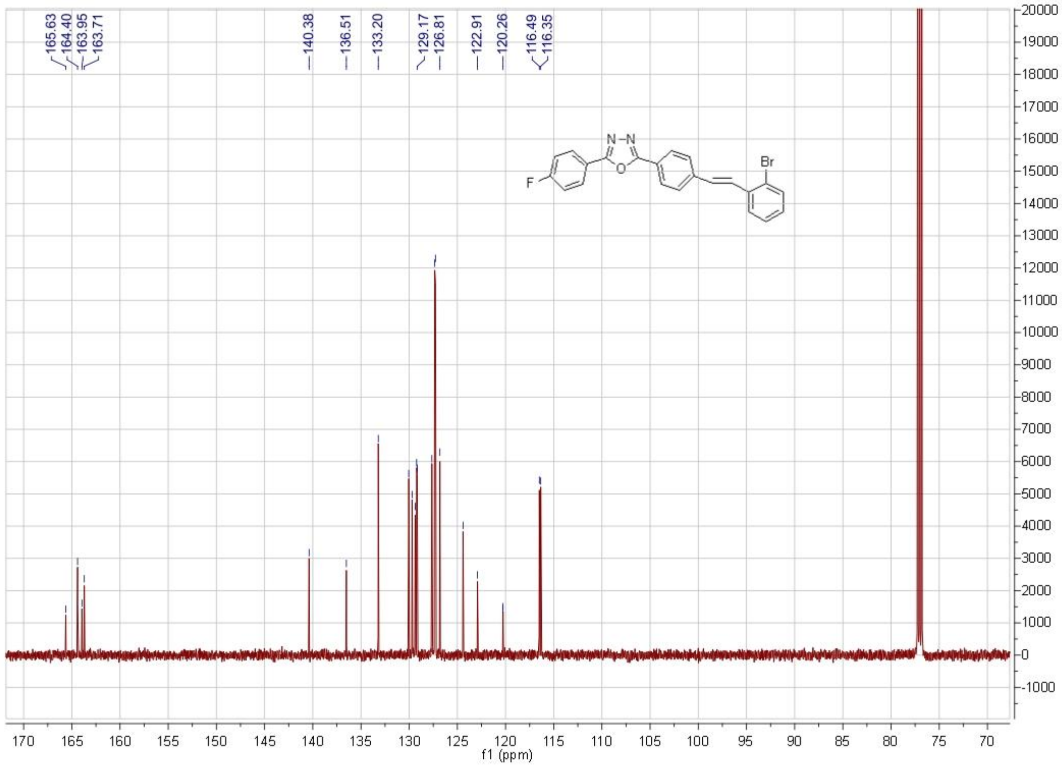

Figure S5-2. ^{13}C NMR spectrum of compound **8**.

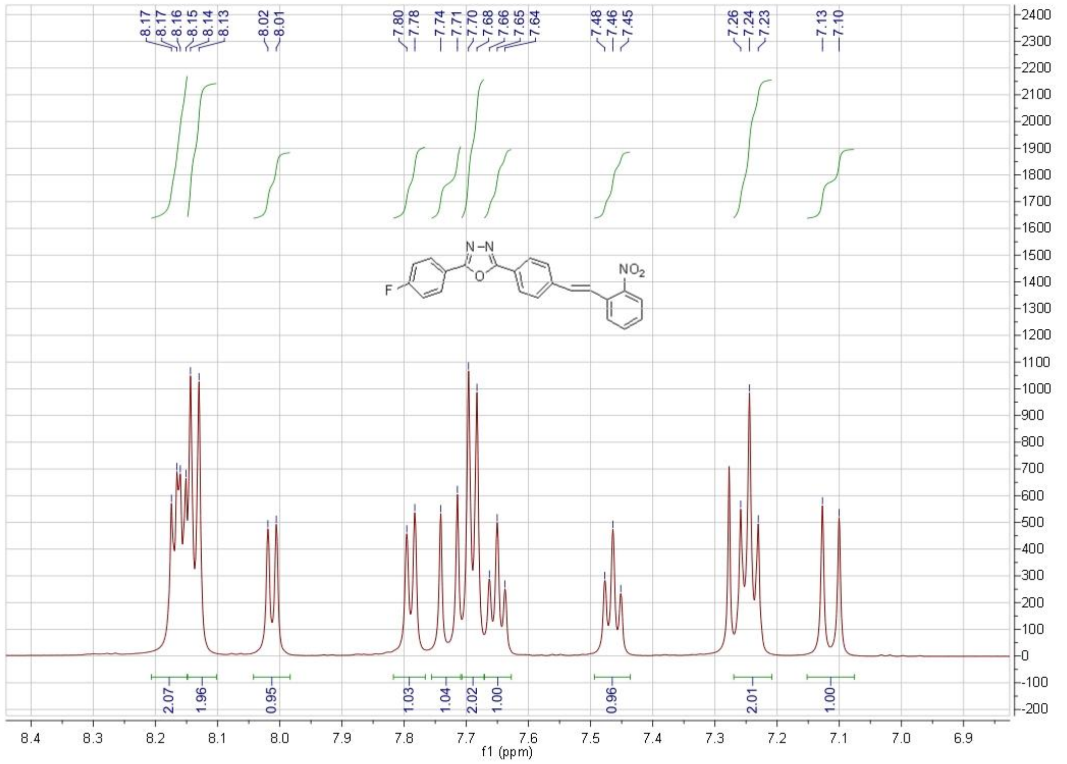

Figure S6-1. ^{1}H NMR spectrum of compound **9**.

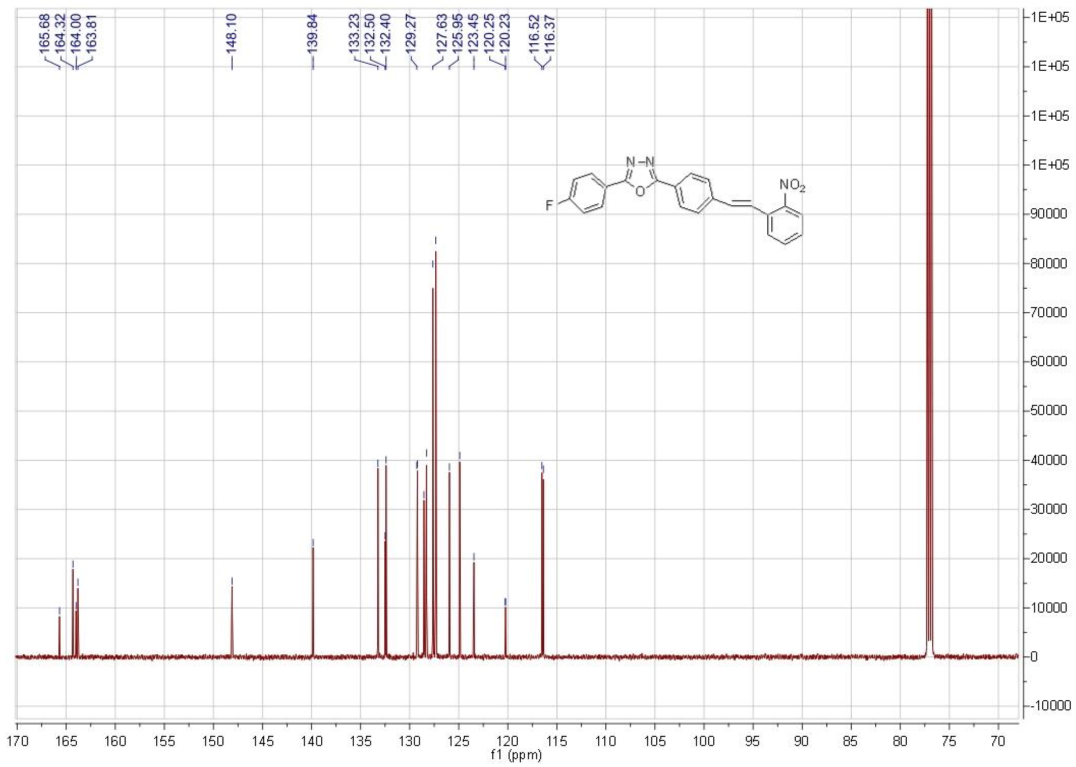

Figure S6-2. ^{13}C NMR spectrum of compound **9**.

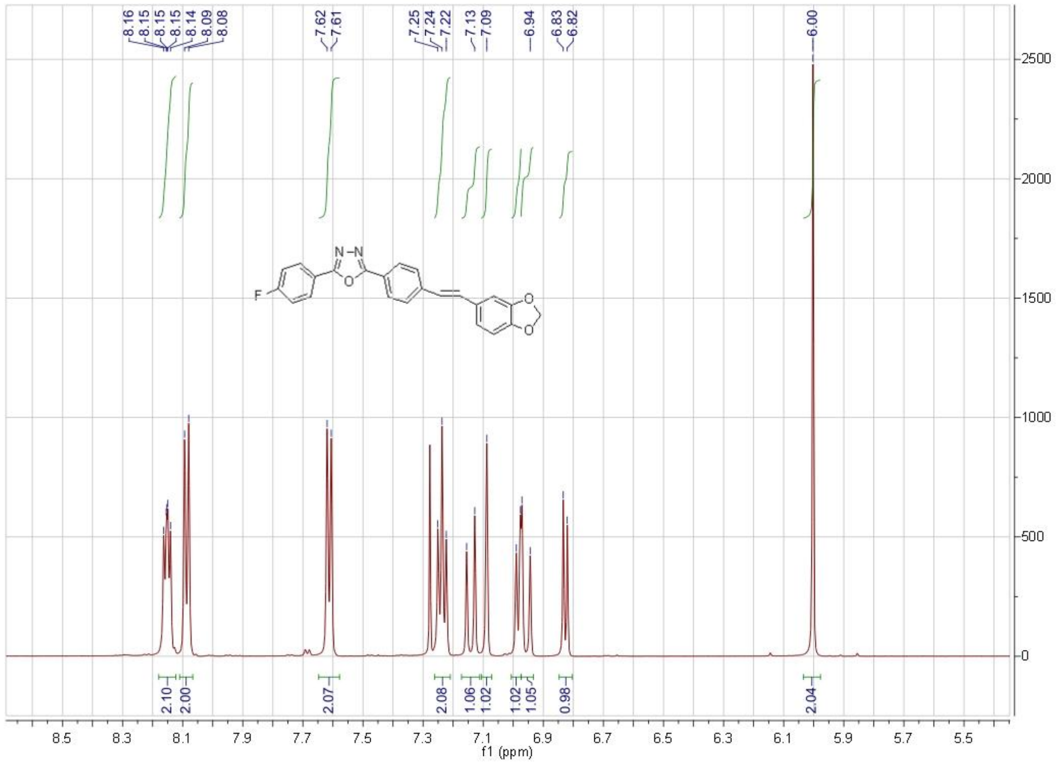

Figure S7-1. ^{1}H NMR spectrum of compound **11**.

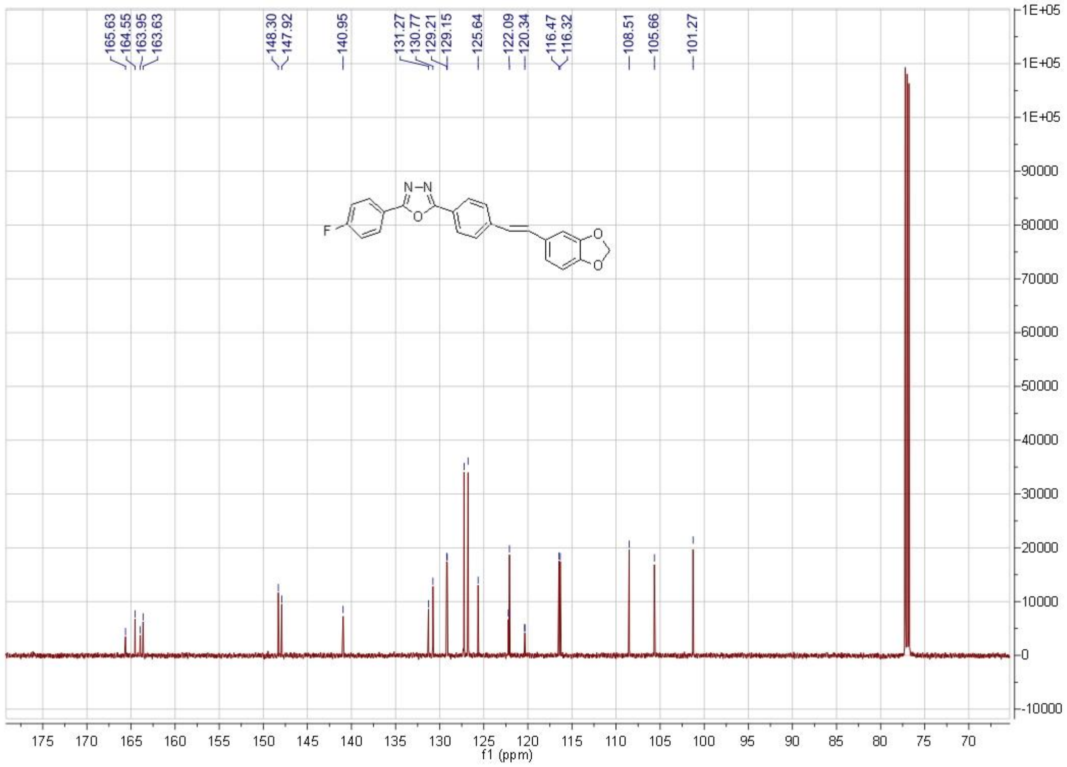

Figure S7-2. ^{13}C NMR spectrum of compound **11**.

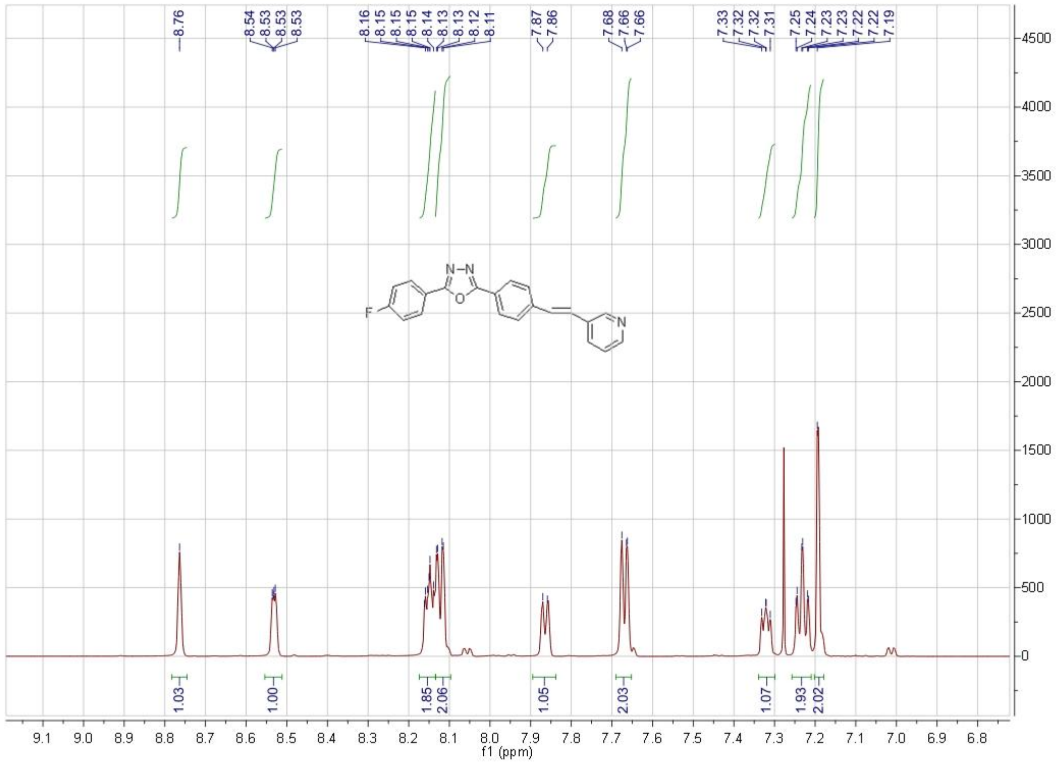

Figure S8-1. 1H NMR spectrum of compound **12**.

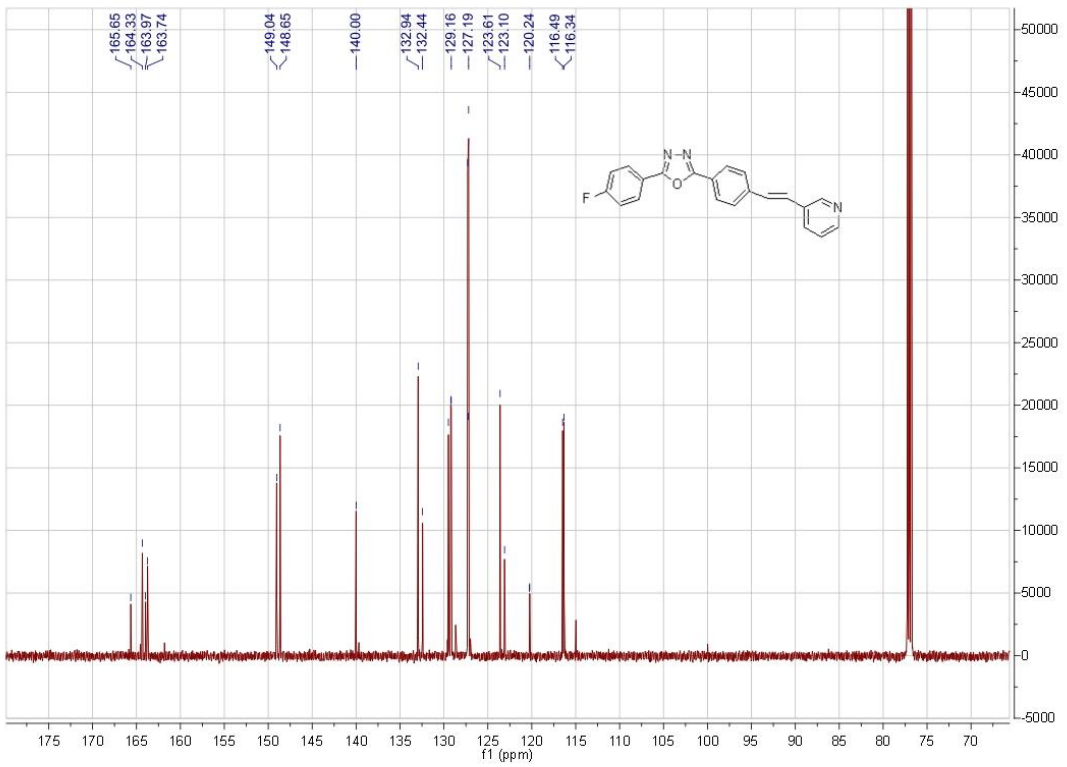

Figure S8-2. 13C NMR spectrum of compound **12**.

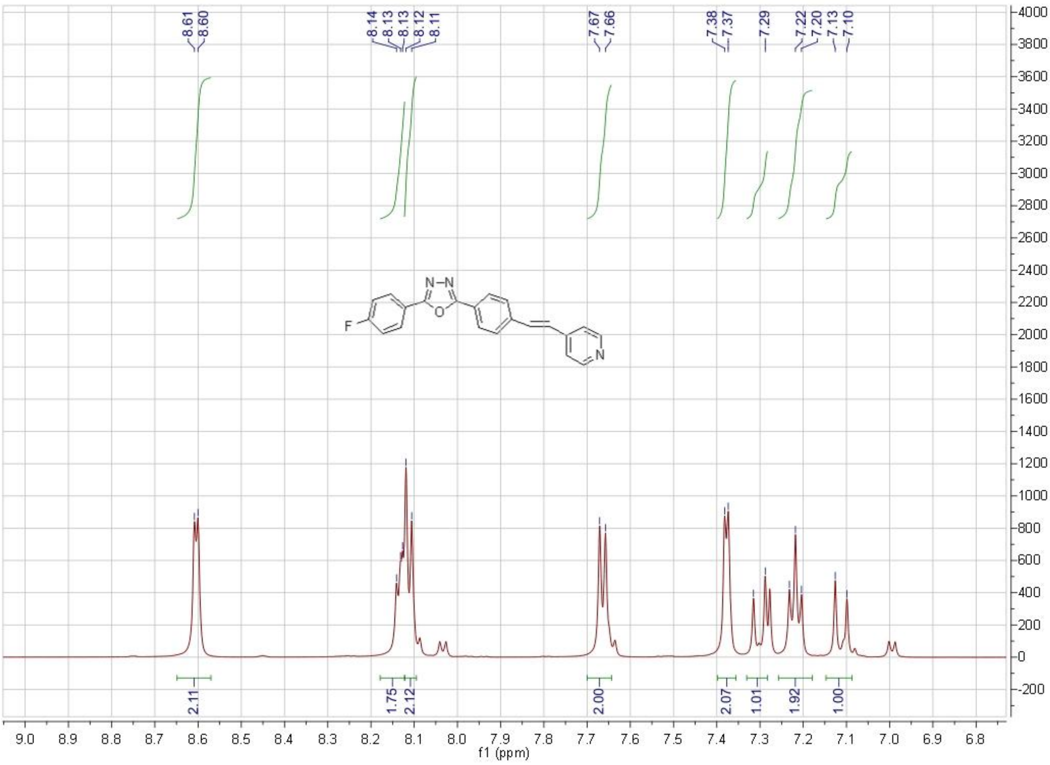

Figure S9-1. ^{1}H NMR spectrum of compound **13**.

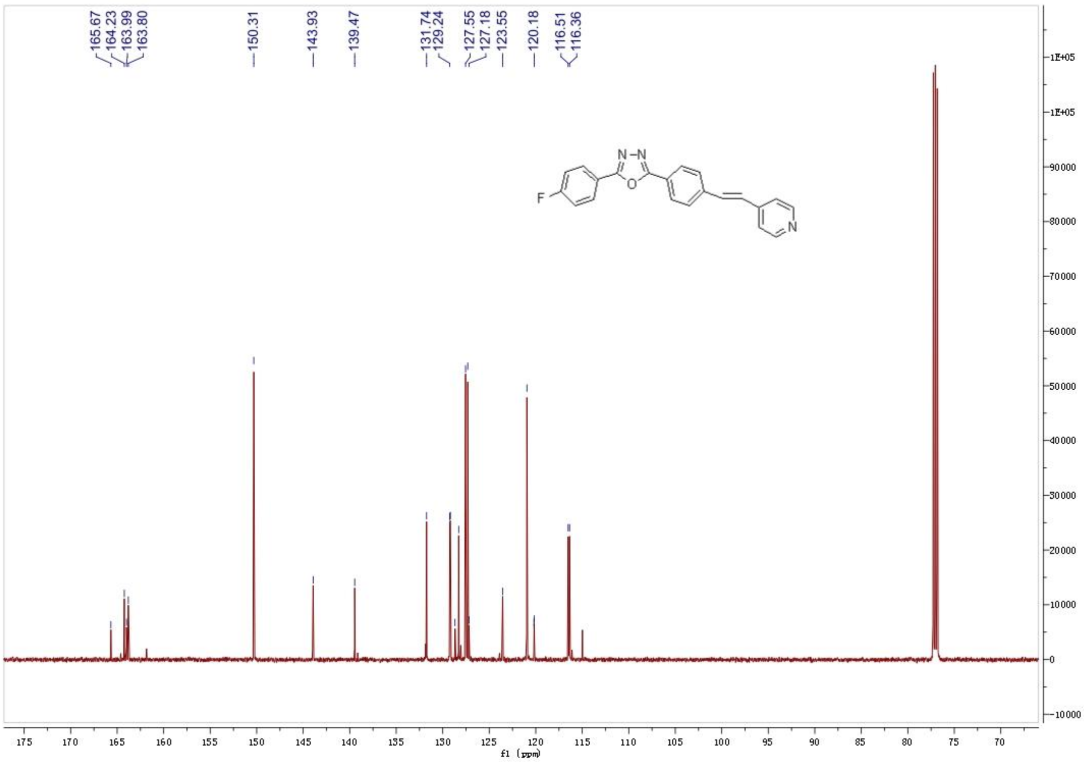

Figure S9-2. ^{13}C NMR spectrum of compound **13**.

References

(1) Jian, W.; He, D.; Xi, P.; Li, X. Synthesis and biological evaluation of novel fluorine-containing stilbene derivatives as fungicidal agents against phytopathogenic fungi. *J. Agric. Food Chem.* **2015**, *63*, 9963–9969.

(2) Laskowski, R. A.; MacArthur, M. W.; Moss, D. S.; Thornton, J. M. PROCHECK: a program to check the stereochemical quality of protein structures. *J. Appl. Crystallogr.* **1993**, *26*, 283−291.
